# Are we truly fighting ableism? Digressions for a complex society

**DOI:** 10.3389/fsoc.2025.1575778

**Published:** 2025-07-30

**Authors:** Lucas Teles da Silva, Dimitri Marques Abramov, Daniel de Freitas Quintanilha

**Affiliations:** Fernandes Figueira National Institute of Women, Children and Adolescent Health, Oswaldo Cruz Foundation, Rio de Janeiro, Brazil

**Keywords:** ableism, Queer (LGBTQIAPN+), complexity, capitalism, diversity and inclusion

## Abstract

Ableism, as a pervasive yet often unchallenged structure of oppression, operates across multiple social domains, shaping perceptions of disability and normalcy. This article interrogates the complexities of ableism through an interdisciplinary framework that integrates complexity theory, Queer theory, and critical disability studies, engaging with the works of Michel Foucault and Georges Canguilhem (among others). Rather than treating ableism as a singular form of discrimination, the study examines its intersections with other oppressive systems, including homophobia, medicalization, and epistemic injustice. By analyzing how blindness, schizophrenia, and paraplegia are socially constructed and regulated, this research highlights how biopolitical and necropolitical mechanisms determine which bodies are deemed valuable, productive, or expendable within neoliberal societies. This framework allows for a deeper understanding of how ableism functions both as a means of control and as a determinant of which lives are considered unworthy of care. Furthermore, by engaging with complexity theory, the article challenges reductionist perspectives that frame disability as an individual deficit rather than as an integral part of human diversity. The implications of this analysis extend beyond theoretical discourse, calling for a reconceptualization of diversity that does not merely accommodate disabled individuals within existing structures but actively deconstructs the epistemological and institutional foundations of ableism. This research contributes to psychological and cultural studies by fostering a critical dialogue on how ableism is reproduced in societal narratives, policies, and everyday interactions. By reframing disability as a site of epistemic and existential richness rather than mere impairment, this article tries to advance a more inclusive understanding of human diversity.

## 1 Introduction

The Cambridge English Dictionary defines ableism as “policies, behaviors, rules, etc. that result in unfair or harmful treatment of disabled people,” as well as “harmful or unfair things that people say, do, or think based on the belief that disabled people are inferior to those without disabilities” (Cambridge University Press and Assessment, 2024).

Such a definition centers on the term “disability,” which the same dictionary describes as “an illness, injury, or condition that makes it difficult for someone to perform certain activities that others can typically do, often in a permanent or long-lasting manner.” In Brazil, ableist practices are criminalized under the “*Lei Brasileira de Inclusão da Pessoa com Deficiência*” (Brazilian Law for the Inclusion of People with Disabilities, LBI), Law No. 13.146/2015. An important distinction to make is that “ableism” encompasses broader societal beliefs and practices, while the LBI specifically addresses discriminatory acts.

Although this is the terminology commonly used in the field, the word itself (disability) implies an inability or absence of ability. However, are people inherently able *a priori*? If disabled, by what standards? Therefore, one may further notice that the terminology used to describe such diverse human conditions reveals that society still fails to prevent inequity, as it labels individuals as simply “disabled” without considering the myriad possibilities of existence that reality can comprise, where different people could flourish once equity is provided. There is a clear contradiction when we claim equity in our relationships, in workspaces, or in our aesthetics, and at the same time define people simply as “not able” (the strict sense of “disability”).

In general, the effects of misusing the word “disabled” and the conceptual contradictions that surround it still extend to norms, practices, and beliefs that marginalize people based on their differences. As we will discuss, society does not provide space for those people given its cultural values and economic paradigms. Would abandoning the word “disabled” confront an entire system of beliefs and practices that make up the contemporary Western world?

Diverse people could be seen as simply different in a complex social system where many human properties that certainly emerged *in nature* are not despised or devalued based on current cultural hegemonic values. “Complex” here is a term adopted in light of its formal and scientific concept: the physical property of a highly informative, integrated, evolutionary, and, who knows, perennial system (Tsallis, 2020). A complex system needs diversity and collectivity, far beyond what we understand as normality and disability. This means a system that thrives on interconnectedness and varied components rather than strict uniformity. Therefore, as we will discuss here, ableism is one of many paths for the collapse of humanity’s future.

Every individual navigates a landscape of limitations and challenges, coupled with unique individual abilities. This essay aims to unpack the misconceptions surrounding the human condition, beginning with the language used to describe it. We will explore the historical roots of current Western thought, informed by positivist and economic paradigms, which are themselves derived from religious frameworks.

## 2 What is normality and where did it come from?

“Normal” is a cultural construction. In Western culture, normality is a classical construct based on the Greek moral and aesthetic ideal of man-kalos *kai agathos* (Nussbaum, 2011). The modern economic paradigm, in turn, added the concept of functionality to the idea of what is normal, deriving this concept from the skills necessary for the work in a system of production (Foucault, 1973; Garland-Thomson, 1997).

The notion of what constitutes “normal” versus “pathological” has long been central to the framing of disability, often with the latter term being used to justify exclusion, marginalization, and discrimination. As Foucault (1973) demonstrated in The Birth of the Clinic (1973), the medical gaze has played a critical role in constructing categories of normality, turning differences into pathologies that can be diagnosed, controlled, and often segregated. In this context, the term “disability” becomes not only a clinical categorization but also a moral and cultural judgment, reflecting broader societal anxieties about deviation from the norm.

### 2.1 From the origins of Western thinking

The historical development of Western thought has deeply shaped the way societies define ability, productivity, and normality. From the medieval period through to the rise of scientific positivism, Western intellectual traditions have constructed paradigms that associate the human condition with predefined norms of functionality, often dictated by economic and social utility. The lens through which humanity is understood has been heavily influenced by economic imperatives, particularly the demand for productivity. This framework, which prioritizes efficiency, labor capacity, and economic contribution, forms the basis for what is considered “normal,” “functional,” or “able” (Davis, 1995).

The contemporary Western culture is idealist, reductionist, and normative (MacIntyre, 1981; Polanyi, 1944), based on a market economy (Harvey, 2005), and it has competitiveness and capitalism as cardinal values or systemic implications to how society works in the end (Bauman, 2000; Harvey, 2005). In the medieval period, societal norms were often informed by theological and religious doctrines that framed disability and difference within a moral and divine context. This perspective, rooted in Christianity, viewed bodily and mental impairments as manifestations of sin, divine punishment, or a moral failing (Foucault, 2009). While this period did not rely on the concepts of “efficiency” and “productivity” in the modern sense, it did conceptualize individuals as either fulfilling or failing to fulfill societal roles, ultimately determining their place within the social order. It is important to note that alternative accounts of pre-capitalist Europe exist, suggesting that disability did not always equate to social exclusion, and that every person, regardless of their very subjective capabilities, might have had a place in society (Slorach, 2015).

### 2.2 The rise of scientific and positivist thought

The dawn of modern science in the Enlightenment and the subsequent rise of positivism further entrenched ideas of normativity through a lens of biological determinism. Thinkers like Auguste Comte played pivotal roles in establishing the frameworks by which human beings and their capacities were measured and compared against a set of idealized standards of functionality. As a consequence, scientists have historically reinforced ableism in their practices, such as treating people with disabilities through demeaning and pejorative terms like “idiot,” “imbecile,” “moron” and “retarded” for people with mental disabilities (Da Silva and Hubbard, 2024).

Ultimately, this ideology evolved into the proposition of the pseudoscience of eugenics by Francis Galton in the late 19th century. Based on an oversimplification of ideas from genetics and natural selection, eugenics proposed that, for the common good of society, the reproduction of “well-born” individuals (e.g., healthy, intelligent, productive) should be promoted, and those who were “defective” should be prohibited from reproducing and passing on their impairments to the next generation (Da Silva and Hubbard, 2024). Although later discredited in its overt forms, eugenics had a pervasive influence on Western biomedical sciences and served as inspiration for discriminatory and violent practices, such as the Holocaust itself. Beyond its historical context, eugenic ideologies contain a disturbing contemporary and/or continuous influence, manifesting in the ongoing institutionalization, forced sterilization, and restricting immigration policies targeting disabled individuals worldwide (e.g., Canada and Australia). Making this ideological stain in humanity, an ongoing issue that needs to be pinpointed (Puar, 2017). Eugenic ideas were especially detrimental to people with disabilities, serving as an allegedly scientific justification for the prejudice and exclusion aimed at those people.

In this context, any deviation from the presumed norm—whether physical or mental—was framed as a deficit. This scientific reductionism paved the way for the categorization and medicalization of human bodies and minds, and for what Foucault (1965) termed the “medical gaze,” which became an essential instrument for both diagnosing and normalizing human existence. In this sense, disability became inherently pathologized: it was defined not by the social or cultural context, but by a deviation from the norms established through these scientific paradigms. As the Enlightenment gave way to industrialization, these pathologized perceptions began to intersect with economic models of productivity and efficiency, which sought to categorize individuals based on their utility within the growing capitalist economies.

Foucault’s (2009) broader historical inquiries and observations, particularly in works such as *History of Madness (2009),* further illuminates how mental health itself, became a political category of deviation, constructed through specific societal practices and institutions. He observes that, madness (for example), became an experience to be medicalized and controlled, while also being something subversive to a singular social fabric. This historical process aligns with the currently western tradition of clustering or pathologizing ‘physical disabilities’ as something deviant from a single norm, excluding and labeling people deemed with such a diagnosis, as people who are considerably unproductive. Thus, mental health as a political canvas over how we deal with certain subjectivities in society, can be a substantial conduit for talking about ableism/disability as something inherently political, albeit defined by very specific logics of power and control.

### 2.3 Capitalism, productivity, and the concept of normality

Marx’s (1867) critique of capitalism, particularly in works such as *Das Kapital* (1867 *Volume 1*), provides a key theoretical framework for understanding how productivity became a defining feature of normality. Marx argued that capitalism reduces human beings to mere commodities whose value is determined by their capacity to produce and contribute to the economy. The emergence of wage labor, where an individual’s worth is measured by their ability to produce goods and services, created a binary: those who could work efficiently and continuously were deemed productive and thus normal, while those unable to contribute to this system—whether due to disability, old age, or other factors—were marginalized as abnormal, dependent, or useless.

This economic paradigm of productivity, reinforced by capitalist values, aligns with the modern conception of functional versus non-functional bodies. In a system where value is determined by labor capacity, the disabled body is often seen as a hindrance to the economic machine (Da Silva and Hubbard, 2024). Marx’s (1867, Volume 1) notion of alienation in the labor process—where workers become estranged from the products of their labor and their human potential—is mirrored in the experience of those labeled as disabled, who often find themselves excluded from productive roles within society. This alienation is not only economic but also social, as it reinforces the idea that disability is inherently linked to an inability to contribute to the capitalist system. Other authors, such as Slorach (2015), Chis (2023), Russell (2001), and Malhotra (2002), can properly highlight to us the inter subjectivities surrounding disability as an adjective defined by capitalistic control, as well. These scholars collectively offer a robust critique of the manner in which capitalism structures the understanding and experience of disability. Slorach (2015) offers a political and historical examination of disability, illustrating its entwinement with the production of capitalist conditions. Chis (2023) expands on this by emphasizing the centrality of disablement to capitalist social relation reproduction and how disability is not an inherent feature but is a process of subjectivation based on economic forces. Russell (2001) and Malhotra (2002) build on this by describing the way that disablement functions within the political economy, suggesting that capitalism creates the very conditions upon which disabled people are constructed and disadvantaged, and upon which they are rendered necessary for its operation. Together, their work goes to explaining that disability, in contrast to the view of it as a medical or individual condition, is a socio-economic construction well-established within, and facilitated by, capitalist production and control systems.

## 3 Who are the “disabled” ones?

### 3.1 Diversity and its political meaning(s)

Society often disregards conditions like blindness, deafness, and some forms of neurodivergence as mere anomalies, failing to recognize them as integral aspects of human diversity within a complex social fabric. This medicalized perspective, deeply rooted in Enlightenment rationality and biomedical discourse, constructs disability as a deviation from an idealized norm rather than acknowledging it as a legitimate mode of existence (Titchkosky, 2007). However, framing these conditions solely in terms of deficit erases their potential contributions to epistemological, cultural, and relational diversity. Consider, for instance, the way childhood is understood: a four-year-old cannot independently navigate the world in the same way as an adult, yet this dependency is not labeled as pathological. Instead, childhood is seen as a developmental stage with unique capacities, perspectives, and needs (Goodley, 2014). Similarly, blindness or autism should not be reduced to a set of impairments; rather, they should be understood as different ways of being that shape how individuals experience, interpret, and contribute to society.

This shift in perspective is essential for moving beyond the dominant framework of ableism, which often seeks to either correct or accommodate disability rather than reimagining social structures to embrace it. This approach aligns closely with the British Social Model of Disability, which views disability as a consequence of societal barriers rather than individual impairment (UPIAS, 1975; Barnes, 2000; Oliver and Barnes, 2012). For instance, Deaf Culture is not simply the reunion of individuals who cannot hear, but a rich linguistic and cultural tradition centered around sign languages and visual–spatial communication (Bauman and Murray, 2014). Likewise, neurodivergent ways of thinking—such as those associated with autism or schizophrenia—challenge conventional notions of reality, perception, and creativity, offering alternative modes of understanding the world (Chapman, 2020). The failure to recognize these as valid and valuable forms of diversity mirrors the way other marginalized identities have been historically framed as deviations from a supposed universal standard. For example, The denial of recognition of indigenous knowledge systems and traditional ecological modes of knowing as valid and deserving expressions of diversity is consonant with the history of non-Christian spiritual practices being framed as the aberrations from some putative universal standard of religious orthodoxy(Smith, 1999; Said, 1978), or, the failure to recognize discrete modes of mobility and sensory experience enacted by people with physical disabilities as valid and preferable forms of diversity is mirrored in the way that non-white racial identities have long been framed as deviations from an assumed universal norm of whiteness and, as a consequence, subjected to systemic oppression (Du Bois, 1903; Fanon, 1952; Mills, 1997).

Diversity, as a social construct, is often interpreted as a medium towards celebrating gender, race, sexuality, and disability. However, this celebratory stance can become frivolous once we acknowledge the exclusionary structures beneath the surface. Ableism, in its multifaceted forms, reveals how diversity initiatives frequently reinforce normative assumptions about bodily and cognitive capacities rather than dismantling systemic barriers (Goodley, 2014). Similarly, the intersection of ableism with homophobia showcases how “queerness” (used here as a broad statement) is frequently underpinned by the exclusionary and dichotomous-based argument of normality (McRuer, 2006). This suggests that the conscientious fight against ableism should be adamantly propelled by the recognition that the concept of “diversity” itself is essentially interwoven by a politically unconscious cultural reality which proposes that certain bodies and subjectivities diverge from a certain norm. For example, diversity initiatives in the labor market that focus on “including” disabled individuals only if they can conform to existing productivity norms, thereby reinforcing the “normal-abnormal” duality rather than challenging the structures that create it.

Within a complex society, power operates through a dispersed (though multifaceted) mechanism, meaning that ableism is at the same time culturally constructed and institutionalized. The medical-industrial complex, for example, does not simply oppress disabled individuals through overt discrimination but also through the production of knowledge that reinforces disability as a defect (Titchkosky, 2007). In a similar vein, the educational system opens itself to diversification, only with the condition that ‘certain subjectivities’ adhere to a predetermined behavioral norm (Meekosha and Shuttleworth, 2009).

The regulation of disability within society cannot be fully understood without engaging with Foucault’s (2003) concept of biopolitics, which describes how modern states exercise power by managing life through mechanisms of surveillance, normalization, and institutional control. Biopolitical power not only seeks to gradually eliminate disability but also to regulate it through medicalization and, ultimately, institutional control. This regulatory logic can be evidenced in the lives of paraplegics who are only integrated into society as long as they can go through very specific forms of “treatment,” such as prosthetics or rehabilitation (Garland-Thomson, 2011).

However, biopolitics is also inextricable from necropolitics, a term coined by Mbembe (2003), which extends Foucault’s framework to analyze how power decides which lives are deemed expendable. Necropolitical structures operate not only through overt violence but also through systemic neglect, as seen in how individuals with schizophrenia or severe disabilities are disproportionately institutionalized, subjected to precarious living conditions, or denied access to care under neoliberal regimes of productivity (Puar, 2017). Ableism then works as both a biopolitical power with ideologically made surveillance regarding who is deserving of control and who is not, interwoven by the necropolitical evaluation of neoliberalism at its core, which is directly responsible for the devaluation and exclusion of certain bodies.

### 3.2 From the normal and the pathological to Queer theory

Western cultural models have long placed individuals who are outside normativized ideals of able-bodiedness into marginal or subordinate positions, thereby reinforcing ableist structures. Queer theory—which is not a settled doctrine but more of a collection of critical lenses—offers a helpful analytical framework to epistemologically critique these structures. Though explicitly engaged with questions of gender and sexuality, Queer theory’s fundamental disruption of normativity and destabilization of identity have meaningful resonance for the analysis of disability.

Critical theory now, particularly Queer-informed theory, increasingly interrogates the notion of the “norm” as a universal or neutral norm. As Butler (1990) made forcefully obvious in Gender Trouble, categories of normalcy are socially constructed (performative) and not natural or based on biology. Butler’s performativity theory—originally formulated about gender—can be applied, with caution, to disability studies.

Performativity in Butler’s theory entails the repeated performative instantiation of norms by lived bodily practices. Transposed to disability, this entails that “disability” is not simply a biological reality but a category constituted by discursive, institutional, and cultural performances. It is important to note, though, that this does not mean that people with disabilities are “performing” disability. Instead, the performance is accomplished through social processes that construct and attribute meaning to disability—medical diagnosis, educational labeling, architectural planning, and policy structures, to name a few.

With that being said, a strictly discursive strategy might miss the embodied materiality of experience. It is therefore important to hold in tension the social construction of disability and the lived life of disabled bodies. A nuanced application of Butler’s theory can illuminate how hegemonic discourses determine the parameters for what is considered “normal” or “pathological.” However, the corporeal and affective existence of disability must be taken into account. This intersectional perspective puts the richness of embodied lives, which are often made invisible by ableist norms and power, front and center.

Rubin's (1984) seminal essay “The Traffic in Women” offers another lens through which we can understand the intersection of disability and normativity. Rubin examines how sexual hierarchies and gender norms are intertwined with social systems of control, including those that manage bodies deemed deviant or non-normative. While Rubin’s focus is primarily on sexual politics, her analysis is also relevant to disability studies because it highlights how bodies are regulated and categorized. Just as certain sexualities are pathologized or stigmatized, so too are certain bodies marked as disabled. Both are part of a larger system of societal regulation that positions them outside the realm of “normal” human experience, reinforcing power dynamics that serve to exclude and devalue these groups.

From a Queer Theory perspective, the category of disability is similarly fluid and contingent. Just as Queer Theory challenges the heteronormative binary of male/female, Queer Disability Studies pushes against the ableist binary of able/disabled. Scholars like McRuer (2006) argue that able-bodiedness itself functions as a kind of normativity, structured similarly to heteronormativity, where individuals who embody the “norm” are considered fully human, while those who do not are marginalized or even erased. McRuer’s concept of “compulsory able-bodiedness” mirrors the work of Queer theorists who have exposed how normative heterosexuality shapes and limits our social possibilities (Butler, 1990). The performance of bodily norms, whether gendered or able-bodied, becomes a site of regulation and restriction, reinforcing the marginalization of those who resist these norms.

To decouple the concepts of normality and pathology from their historically entrenched meanings is to imagine new ways of being and relating that are not confined to binary distinctions. Queer Theory’s focus on fluidity, non-normativity, and resistance to fixed identities offers a framework for rethinking disability. Rather than pathologizing diverse bodies and minds, Queer Theory invites us to embrace the multiplicity of human experiences, rejecting the assumption that there is a singular, ideal way to be human. By using a Queer lens to analyze disability, we can better understand the dynamic, evolving nature of the human condition and the potential for creating more inclusive, equitable societies that honor difference rather than marginalizing it. “Everyone has a part of their life that causes them shame, that they do not show to others, and that affects their way of relating to others. The closet is a place of nonexistence, a place where life can be seen but cannot be touched” (Portero, 2024).

### 3.3 Ableism and the neoliberal productivity paradigm

From industrialization and throughout capitalism, with its basis on the notions of individualism and productivity, discrimination and exclusion of those deemed not fit for the system have been the norm, especially people with disabilities (Mannor and Needham, 2024). In the late 20th and early 21st centuries, the dominance of neoliberal economic frameworks further entrenched this understanding of normality and ability. Neoliberalism, with its emphasis on individualism, market-driven policies, and the devaluation of the social safety net, has exacerbated the marginalization of diverse individuals. Neoliberal thought, which prizes personal autonomy and self-sufficiency, often equates productivity with value. Within this framework, individuals with disabilities are frequently cast as economically nonviable and, therefore, deviant from the normative ideal of a productive, autonomous citizen (Rose, 1999).

It is a particularly difficult task to harmonize our culture with humanist values regarding diversity (if possible). Moving away from a neoliberal approach that partially includes only worthy/working people with disabilities while also disrupting other ableist representations of disability requires going beyond including more people with disabilities within the exploitative and individualized social relations of neoliberalism. That is, challenging the contemporary biopolitics of “disability” requires more than access to education, employment, or social lives, but rather requires changing the conditions, practices, and discourses that surround and produce social disability (Fritsch, 2015). Real transformation demands a fundamental shift, and this includes revolutionizing how we imagine and create our subjectivities.

### 3.4 A Brazilian legal framework

In the Brazilian context, the Lei Brasileira de Inclusão (LBI) represents a groundbreaking legal instrument that criminalizes disability-based discrimination, ensuring the possibility at least, for a broadened and healthy public space for people diagnosed with any disability. However, empirical studies point to substantial political gaps. For example, in schools (private or public), there is a lack of proper staff to attend to children diagnosed with a ‘disability, resulting in a significant number of kids dropping out of school early on. As Nogueira and Santos (2022) argue, the LBI is undermined by the ongoing political structures that neglect or deconstruct the material reality of such inequalities in our society. This disparity highlights the importance of analyzing this issue not only on theoretical grounds, but also considering the political practices of the Brazilian society, which is absorbed by colonial problems and structural inequality.

Brazilian scholarship provides a critical framework to conceptualize ableism independently of borrowed theoretical schemes, frequently linking it to past and present socio-economic inequalities. For instance, researchers like Fritsch (2015) examine the neoliberal biopolitics of disability in Brazil to show how commodification of life and labor under neoliberalism plays an important role in determining who gets to be “able” and who is left out, and in the process, they discover that inclusion strictly depends on being productive and independent. Furthermore, evidence from scholars such as Meekosha (2011), even though from a general decolonial perspective, strongly echoes the Brazilian situation by emphasizing how ableist architectures that are reproduced by colonial legacies shape public policies and social opinion regarding disability. This decolonial critical vision, shared widely in the broader Latin American disability studies, argues that ableism is inherently bound to intersectional oppressions like race, class, and gender and necessitates localized analyses sensitive to the concrete structural and historical injustices of the Global South.

## 4 Complexity and diversity intertwined

### 4.1 What makes up complexity?

In the contemporary discourse on diversity, the concept of complexity plays a pivotal role in understanding the intricate tapestry of human experiences. In Physics, complex systems—whether social, biological, or ecological—are not merely the sum of their parts but are characterized by interdependencies, nonlinear relationships, and emergent properties (Miller and Page, 2007).

Morin (2017) synthetically presents to us the possibility of understanding “complexity”: “that which is woven together.” Complexity, far from being a quality of Nature, is nowadays a new epistemology of human reason, set alongside classical, Cartesian, and positivist scientific thought: a large part of the world is only intelligible in terms of complex thinking.

We identify a complex system wherein we observe a group of diverse individuals (individual diversity) that are indeterminate (in chaotic configurations, states, and behavior) from which, surprisingly, collective organization processes emerge, without central control or *a priori* design. Self-organization at an order level above individuals is possible because the fact is that these individuals are also intrinsically related or connected to each other. Diversity promotes greater possibilities for the emergence of new states. And connectivity/correlation allows for the resonance and amplification of local change to the global level. Thus, in a paraphrase of Morin, complexity is the activity of diverse actors who co-create multiple possible common realities.

A universe devoid of diversity would be compared to a crystal, where every individual is exactly replicated to form a monotonous macro-structure and which is incapable of evolving. In the absence of diversity, there can be no emergence of novelty (even from the physical point of view!), no self-organization. There would be no life and its evolution arising in a pluripotent universe made up of billions of species, no human mind, and not even society.

By extension, complex thinking assumes the characteristics of complexity. Complex thinking enables plurality within a logic where “AND” takes the place of “OR,” where dichotomy and monovalence are the exceptions instead of the rules. In systematizing old reductionist scientific thinking, we brought ancient medieval moral teachings into modernity. Previously, something was right or wrong for purely moral and metaphysical reasons; now we decide something as right or wrong for scientific reasons, by the logic of reductionist thinking. Reductionist scientific thought has solved countless problems and brought unimaginable technological advancements four centuries. But answers to ancestral questions such as “what is life” or even “who are we” lie outside of normal scientific thought. Our culture imposed a secular life. So Western man thinks in a way incongruent with the reality of the world. The complexity science today proves that non-complex processes are the exception instead of the rule (Tsallis, 2023; Gell-Mann and Tsallis, 2004), so the Universe, from astrophysics to cultural evolution, evolves under the paradigm of complexity.

Far beyond physics and Biology (Maturana and Varela, 1987; Capra, 1996), Philosophy, through Spinoza’s Ethics and his theory of affects (Peixoto, 2016), as well as the works of Morin (2017), has presented complexity as an interdisciplinary paradigm.

### 4.2 What can be said about ableism through the lens of the complexity paradigm?

The works of Morin (2017) have been leading philosophical inquiries into the field of complex thought and its implications, contributing significantly to the creation and consolidation of the complexity paradigm. Morin’s theory understands complexity not as an answer, but as a challenge for our worldview and knowledge. It conceives complexity as composed by principles, some of which we can highlight and use as tools to shed light onto the problems concerning ableism in an attempt to explore and propose new insights. Such an approach is in line with Morin’s view of the complexity paradigm as having its essence in the tendency to build relations.

The recursive principle states that a core trait of complexity is the capacity of a being to create the conditions for its existence—autocausation. Recursion is a defining characteristic of living beings (Maturana and Varela, 1987), but is also observed in cultures and cultural practices. For example, the discrimination that people labeled as disabled suffer plays an important role in keeping those people away from socially valued spaces—education, work, media, etc.—, reinforcing ideas of them being incapable of occupying those spaces due to the resulting lack of representation, ultimately creating a feedback on discrimination itself. Accordingly, labeling those people as disabled reproduces the idea that there is a norm—being able—from which some people diverge, which keeps this idea alive. There may be many other examples of ways through which ableism maintains itself, but the fact is that the only way to stop its recursion is to block the feedback cycle—for example, opposing the use of discriminatory language.

The dialogic principle states the urge for dialogue between different ideas and people for the establishment of complexity. As such, the complexity paradigm embraces the employment of fuzzy logic; thus, different propositions are not seen as inevitably opposing or mutually exclusive, but as possibly connected and complementary. We propose ableism as a product of a worldview that lacks complexity and, therefore, dialogue. It is characteristic of a simplistic way of thought to try and reduce, disjoin, and oversimplify complex concepts as an attempt to better understand them, but the consequence of this approach is often the opposite, leading to a poor and reductionist view (Morin, 2008).

Contrary to the principles of the complexity paradigm, ableism poses itself as a conditioning principle; that is, a principle that conditions (limits and regulates) thought, hindering people from perceiving things that are outside its scope. As such, ableism as a conditioning principle produces simplified and reductionist ideas about human existence, limiting the concept of being human to a bundle of capacities and dehumanizing those who do not fulfill them (Reynolds, 2021). Human existence is singular and varied simultaneously. Capacities are part of what people are, but people are more than the sum of their parts. However, only a way of thinking that comprises complexity can dialogue with ideas like that without the need to simplify them (Morin, 2008).

We propose a dialogue between the ideas of ableness and disableness, as they do not exist in absolute. No one is able or disabled in everything. Indeed, every person encompasses both abilities and disabilities in them, interwoven in complex ways that make every person unique. In fact, some people deemed as disabled might even report valuable aspects of the condition they experience—the case of people with attention deficit hyperactivity, which is considered a mental disorder (and, therefore, inherently a lack of functionality), although a majority of those people report positive characteristics of having the condition (Schippers et al., 2022).

In other words, ability is a concept that is difficult to define with clear and precise borders. Therefore, we propose that ability and disability should be seen as a continuum that is constructed amongst the social environment and is a characteristic of humanity as a whole, not an aspect of some “disabled” individuals. This does not imply making people with disabilities and their daily struggles invisible. On the contrary, it is about showing that their struggles derive from beliefs and practices of a society that is *unable to include* them, not inherently from their differences. This shift from the notion of disability as an individual trait to the comprehension of it as a contextual factor that emerges from the interaction between the individual and the physical and social environment is fundamental for a more complex and effective confrontation of ableism (Reynolds, 2021).

### 4.3 What is a complex world made with diverse people?

The political, cultural, and economic understanding of the problem of ableism and the importance of human diversity for a prosperous and healthy society needs to undergo the paradigm of complexity. Thus, this perspective invites us to reconsider the traditional binary classifications that often underpin ableist narratives, framing disability as a deficit rather than a unique facet of human diversity. Diversity in humanity, in its broadest sense, encompasses variations in race, ethnicity, gender, age, sexual orientation, and ability, reflecting the multifaceted nature of human existence (Rosenblum and Travis, 2000). Acknowledging this diversity requires an understanding that each individual possesses a unique set of strengths and challenges, shaped by an interplay of personal, social, and environmental factors.

Moreover, a complex worldview challenges the prevailing paradigms of reductionism often found in Western thought. Reductionism, which seeks to understand phenomena by breaking them down into their constituent parts, can obscure the holistic nature of the world, particularly the human conditions (Capra, 1996). As Canguilhem (2008) argues, the norm should not be viewed as an absolute standard; rather, it is essential to recognize the dynamic interactions that define health and illness.

## 5 Discussion

In exploring the intersections of ableism, normativity, and diversity, this essay has examined how societal frameworks of normality have historically marginalized those who deviate from the idealized “able-bodied” and “productive” standards. Drawing on the insights of diverse theories, it becomes evident that human diversity—whether in terms of race, gender, ability, or other social categories—is not a mere additive quality, but an emergent property of dynamic, independent systems. This holistic view challenges the reductionist paradigms that have dominated Western thought since the medieval period and continue to shape our understanding of diversity as disability. As Canguilhem (2008) and Foucault (2006) suggest, the distinction between the normal and the pathological is socially constructed and serves as a tool for regulating bodies and behaviors by societal needs, often in ways that marginalize those who fail to conform.

The historical shift from medieval religious doctrines to scientific positivism, coupled with the rise of capitalist and neoliberal economic frameworks, has entrenched the valorization of productivity and efficiency, further solidifying ableism as a central axis of social exclusion. As Marx (1867) and Fritsch (2015) have shown, the commodification of human labor in capitalist societies has rendered non-productive bodies—whether disabled, elderly, or otherwise outside the economic machine—as disposable or inferior. The neoliberal model exacerbates this by framing individuals with disabilities as liabilities, measuring their worth through a lens of economic viability. Thus, the challenge is not merely to provide access to education or employment, but to radically transform the structures and narratives that produce and sustain such exclusionary systems.

Queer Theory, particularly as articulated by Butler (1990) and Rubin (1984), offers a transformative framework for rethinking the categories of normality and pathology. By extending the theory of performativity to disability, we can reject the binary logic that constrains both gender and ability. As McRuer (2006) points out, compulsory able-bodiedness mirrors the mechanisms of heteronormativity, both of which function to marginalize those who resist conformity. A Queer lens, therefore, not only illuminates the fluidity and diversity of human experience but also calls for a rejection of fixed identities and the rigid classifications that undergird ableism.

The complexity paradigm is a theoretical tool that enables us to explore new insights into various themes, including ableism and diversity (Morin, 2008). In this paper, we have proposed more complex ways of understanding diversity which go beyond the simple inclusion of people who fall outside of what is considered “normal”; on the contrary, we challenge the normal-abnormal binary by exposing how it is a social construct and analysing how diversity is characteristic of humanity itself, not a particularity of some deviant individuals. Embracing a complex understanding of diversity aligns with principles of intersectionality, which emphasize that identities and experiences are shaped by multiple, overlapping social categories (Crenshaw, 1989). This approach allows for a nuanced exploration of how ableism intersects with other forms of discrimination, revealing that the experience of disability is not monolithic but rather shaped by various factors, including race, gender, and socioeconomic status (Shakespeare, 2006). In this context, the term “diversity” transcends mere representation; it becomes a lens through which we can examine the rich tapestry of human experience that exists beyond conventional norms (Schneider, 2006).

Based on our earlier discussion of pathologization and the construction of normality in the past, it is easy to understand how ableism arises in relation to the social understanding of mental health. Despite significant technological and economic progress, affluent societies manifest out-of-proportion elevated rates of anxiety, depression, and other related mental illness (WHO, 2023). These cultures, typically organized around concepts such as individualism and neoliberal forms of progress and productivity, are thoroughly shaped by the very normalizing gaze Foucault was arguing against. In these cultures, mental distress is often seen as a subjective failure, a ‘disability’ in itself, rather than a potential consequence of pressures of the system to be constantly productive and conform to an ‘able’ standard. This serves to stigmatize the non-completive, ableism being instilled into the very essence of modern life (Abramov and Peixoto, 2022). These situations show that mental distress is not only defined by clinical diagnosis but also by a culturally formed manner of conceptualizing human subjectivity, a sign of a society’s inability to embrace polymorphous forms of existence. In order to overcome ableism, therefore, is to pay closer attention to how political stances and culturally derived attitudes educate us about the possibilities of the human body both subjectively and objectively.

Ultimately, this paper invites a reimagining of diversity as part of the broader human condition—a diverse and evolving spectrum of lived experiences, rather than an inherently pathological deviation from the norm. An inclusive and diverse society is necessary for a complex and healthy life. Embracing this complexity and the intersectional nature of identities offers the potential for a more equitable society, one where difference is not merely tolerated but celebrated. In dismantling the closets of ableism, we open the possibility for a future where all forms of human existence can be seen, lived, and celebrated. By answering the title of this article with a historically and epistemologically based reflection, we got to recognize how far we have come and how far we still need to go. We must revolutionize our subjectivities for diversity to be the gateway to our future as a society.
